# Hyperekplexia: Unveiling a Rare Neurological Condition With a Treatable Solution

**DOI:** 10.7759/cureus.61770

**Published:** 2024-06-05

**Authors:** Nisha R Aglave, Rachana A Sontakke, Chandrakant Bokade, Kush Jhunjhunwala

**Affiliations:** 1 Pediatrics, Datta Meghe Medical College, Datta Meghe Institute of Higher Education and Research (DU), Nagpur, IND; 2 Pediatrics, Narendra Kumar Prasadrao (NKP) Salve Institute of Medical Sciences and Research Center and Lata Mangeshkar Hospital, Nagpur, IND

**Keywords:** clonazepam, glycine, stiffness, startle response, hyperekplexia

## Abstract

Hyperekplexia (HPX) is a rare hereditary disorder characterized by an exaggerated startle reflex and neonatal hypertonia. It exhibits both autosomal dominant and autosomal recessive inheritance patterns, depending on the gene involved. It could be a fatal neurogenetic disorder, but it is treatable. We reported a nine-month-old female child with mild gross motor delay, an exaggerated startle reflex, and multiple episodes of transient hypertonia. Neurological and electrophysiological investigations and clinical presentation suggested the diagnosis of hereditary HPX. The child showed a good response to oral clonazepam, with a reduced frequency of such episodes and attainment of age-specific milestones.

## Introduction

Hereditary hyperekplexia (HPX) is an inherited neuronal disorder that is characterized by pronounced startle responses and stiffness to unforeseen sensory or acoustic stimuli [1]. Initially described in 1958 by Kirstein and Silfverskiold as the 'emotionally precipitated drop seizure,' various terms such as 'hereditary stiff-baby syndrome,' 'congenital stiff-man syndrome,' and 'hyperexplexia' were employed before the terminology HPX was coined by Gastaut and Villeneuve [2]. The symptoms include an exaggerated startle reflex in the form of flexor spasm of the trunk and typical eye blinking, which have been present since birth and sometimes even intrauterine. Tapping the tip of the nose, nose ridge, glabella, or upper lip can precipitate the startle reflex in such children, referred to as the head-retraction reflex (HRR) [3]. There is a lack of habituation to excessive startle and prolonged stiffening in neonates and young infants, which is unlike the physiological startle [4]. HPX neonates and infants experience episodes of apnea, presenting with periodic stiffness and cyanosis, which usually resolve over infancy. If not addressed, this condition may be linked to sudden infant death due to apnea, aspiration pneumonia, and severe injuries, resulting in the impairment of mobility caused by frequent falls [5]. Glycinergic neurotransmission serves as a pivotal inhibitory factor in the central nervous system (CNS); disruptions in this mechanism can give rise to HPX, a startle disorder observed in both pediatric and adult populations. The pathogenesis of HPX is closely associated with the inhibitory glycine receptor (GlyR), specifically the postsynaptic α (1)-subunit encoded by glycine receptor alpha 1 (GLRA1). Additionally, the presynaptic glycine transporter, referred to as solute carrier family 6 member 5 (SLC6A5) or glycine transporter (GlyT2), represents another crucial gene implicated in the development of this disorder [6]. Clonazepam and Vigabatrin are the two drugs studied in HPX. Clonazepam is effective as it potentiates the neurotransmitter γ-aminobutyric acid (GABA) by some unknown mechanism. Vigabatrin inhibits the GABA catabolic enzyme GABA-transaminase, thus increasing GABA [7].

## Case presentation

This case report is about a nine-month-old female infant born with second-degree consanguinity. She was delivered full term via normal vaginal delivery, with a birth weight of 2900 grams. She was brought in with complaints of intermittent episodes of abnormal body movements, characterized by the tightening of all four limbs lasting for 20 seconds, which had been present since birth. These episodes also occurred during sleep and were sometimes associated with a bluish discoloration of the face. The episodes did not result in any loss of consciousness or lethargy. During admission, a drop in saturation was observed. The mother noted that the baby exhibited startle responses even with minimal tactile or auditory stimuli.

Her developmental quotient in the gross motor domain was 60%, while other domains like fine motor, language, and social were normal for her age. On examination, the baby was euthermic with no facial dysmorphism and a heart rate of 128/minute, a respiratory rate of 28/minute, and a blood pressure of 94/50 mmHg. An umbilical hernia was present. During the central nervous system examination, there was transient hypertonia in all four limbs, and deep tendon reflexes were exaggerated with extensor plantars, which was normal for age. Further neurological examination revealed that gently tapping the bridge of the nose with a finger in a calm child, elicited an exaggerated startle reflex, accompanied by symmetric myoclonic jerking of limbs. Notably, this response did not habituate despite repeated stimulation at one-second intervals. Blood investigations were normal for her age (Table 1).﻿

**Table 1:** Blood investigations on admission

Blood investigation	Observed value	Age related reference range
Hemoglobin (Hb)	10.1 gm/dL	11.5-14.2 gm/dL
White blood cells (WBC)	13,320/cubic mm	5000-19000/cubic mm
Platelet counts	476,000/cubic mm	150,000-450,000/cubic mm
Serum Calcium level	9.4 mg/dL	9-11 mg/dL
Ionic calcium	4.7 mg/dL	3.7-5.9 mg/dL
Serum Magnesium level	1.8 mg/dL	1.6-2.4 mg/dL

**Figure 1:**
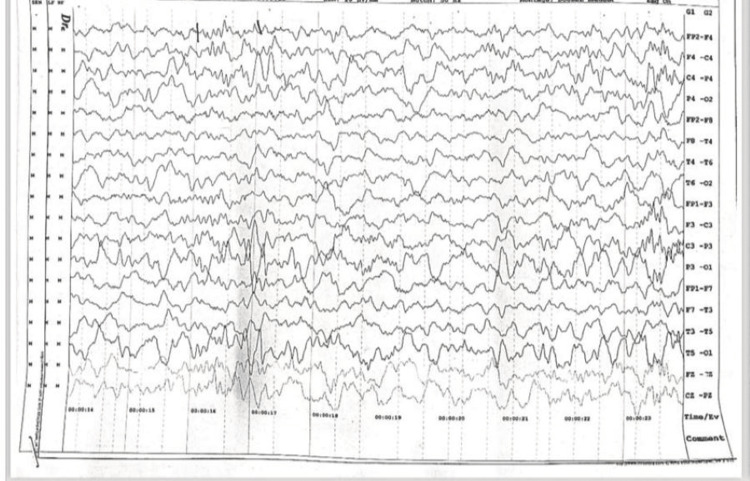
Electroencephalography EEG: Electroencephalography showing generalized epileptiform activity.

The electroencephalogram was suggestive of generalized epileptiform activity, which was not localized to any lobe (Figure 1).

**Figure 2:**
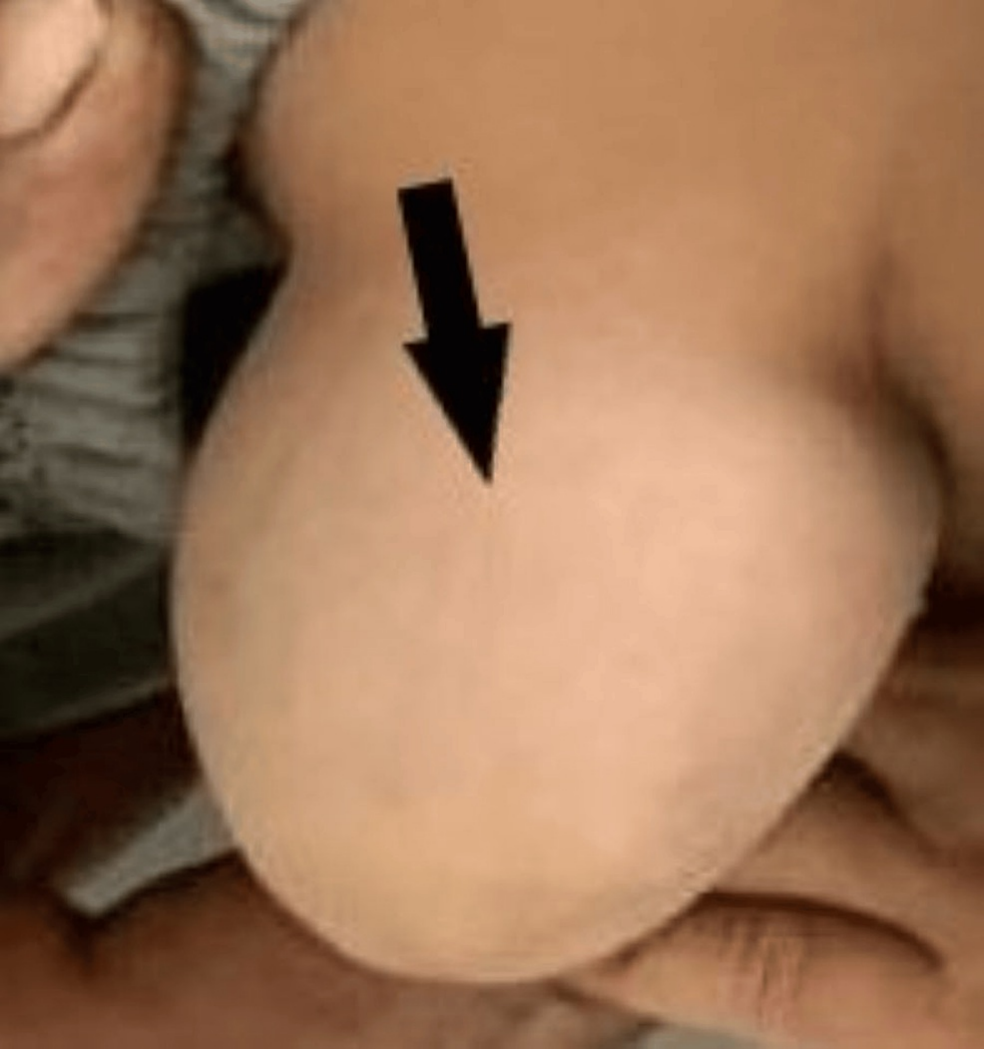
Umbilical hernia in a case of hyperekplexia Black arrow showing a large umbilical hernia

Magnetic resonance imaging revealed normal findings. Ophthalmic evaluation showed no evidence of cherry-red spots or cataracts. The otoacoustic emission was bilateral. Ultrasonography of the abdomen confirmed the umbilical hernia of size 5.5 x 6.3 centimeters (Figure 2).

These events were treated as seizures, and a working diagnosis of neonatal epileptic encephalopathy was established. She was started on multiple antiepileptic medications, such as phenobarbitone and levetiracetam, along with a trial of pyridoxine, folinic acid, and biotin. Seizure-like activity and an exaggerated startle were not controlled with these medications. Clinical presentation and neurological findings suggested the probable diagnosis of hyperekplexia. After researching the literature for the treatment of hyperekplexia, we started the patient on clonazepam at 0.05 mg/kg/dose. Over seven to ten days after starting treatment, the child responded to treatment, and the frequency of startles and tightening of limbs was reduced. Counseling regarding the need for genetic studies was conducted, but due to financial constraints, parents were not ready for further evaluation. To prevent episodes of hypoxia due to spasms at the time of tightening of limbs, parents were counseled and trained to perform the Vigevano maneuver. On follow-up after a month, the baby achieved gross motor milestones corresponding to her age and was free of any events.

## Discussion

Hyperekplexia is a rare, pharmacoresponsive neurological condition that mimics seizures. The prevalence rate of hyperekplexia is less than 1 per 1,000,000. The startle reflex, a reticular and cortical reflex, is a protective mechanism characterized by sudden jerky movements in response to auditory or tactile stimuli. The pathological state is termed hyperekplexia [8]. When there is an exaggerated startle reflex, it interferes with normal activity and may cause life-threatening apnea, rigidity, and hypertonia, which is mostly truncal, and these episodes are attenuated in sleep. Also, such episodes decrease in frequency over infancy [9]. Our child has developed stiffness in the body and episodes of cyanosis since the neonatal period.

Other complications associated with hyperekplexia are attacks of tonic neonatal cyanosis, which are due to generalized stiffness. Sudden infant death syndromes are also associated with unrecognized hyperekplexia [10]. A high prevalence of congenital dislocation of the hip, inguinal, umbilical, or epigastric hernias, and paralytic ileus is seen in hyperekplexic children [11]. This child had an umbilical hernia, which was surgically corrected on follow-up at one year of age.

Exaggerated startle reflex or hyperekplexia could be acquired or hereditary. Hyperekplexia is seen in certain acquired conditions, like pontine involvement, infections like Clostridium tetani, strychnine poisoning, and some autoimmune conditions with glycine receptor antibodies. Hereditary hyperekplexia is caused by mutations in various genes that cause defects in inhibitory glycinergic neurotransmission. The identified genes encompass glycine receptor alpha 1 (GLRA1), solute carrier family 6 member 5 (SLC6A5), glycine receptor beta (GLRB), guanine nucleotide exchange factor 9 (ARHGEF9), and gephyrin (GPHN). Notably, GLRA1 stands out as the predominant pathogenic gene in hereditary hyperekplexia, the presence of exaggerated head retraction reflexes in response to nose tapping signals an exaggeration in brain stem reflexes, offering a crucial diagnostic clue. This aspect should be incorporated into the examination of suspected cases for a comprehensive evaluation [3]. A family history of similar complaints points towards an autosomal dominant pattern of inheritance. Usually, serum electrolytes and other biochemical investigations remain normal. Neuroimaging and electroencephalography (EEG) also remain normal in these patients. EEG waveforms are studied based on their location, amplitude, frequency, morphology, continuity (rhythmic, intermittent, or continuous), synchrony, symmetry, and reactivity. The most commonly studied waveforms are delta (0.5 to 4 Hz), theta (4 to 7 Hz), alpha (8 to 12 Hz), sigma (12 to 16 Hz), and beta (13 to 30 Hz). The delta wave is seen physiologically in deep sleep, while its presence in the awake state indicates focal cerebral dysfunction or generalized encephalopathy. Focal cerebral dysfunction is also suggested by the presence of theta waves in the awake state; this wave is normally present during drowsiness and during the early stages of sleep. Alpha waves are best seen with eyes closed in an awake state; they are attenuated by eye-opening. In generalized cerebral dysfunction, there is a slowing of alpha activity. Beta activity is the most frequently seen rhythm in normal adults and children. It often increases during drowsiness and the early stages of sleep; sedative medications like barbiturates, choral hydrates, and benzodiazepines also increase its amplitude. Focal, regional, or hemispheric attenuation of beta can occur with a cortical injury, malformations, or subdural, epidural, or subgaleal fluid collections [12]. Careful observations of attacks of cyanosis and seizure-like symptoms can help to differentiate them from true seizures. Episodes of hypertonicity accompanied by cyanosis can be halted by a simple intervention called the Vigevano maneuver. This maneuver involves flexing the head and legs towards the trunk [13].

Hyperekplexia is a treatable condition using pharmacotherapy. Clonazepam and vigabatrin are the two drugs found to be effective for hyperekplexia [7]. Clonazepam enhances the function of GABA-gated chloride channels, compensating for defects in genes related to glycine-gated chloride channel function. It is the drug of choice in hyperekplexia [14]. Infants commonly need higher doses of clonazepam (0.1-0.2 mg/kg/day) to effectively diminish episodes of startling and life-threatening events. This regimen contributes to a notable decrease in the associated morbidities and mortalities related to the condition [15]. Our child required an initial dose of 0.05 mg/kg/day, which was increased to 0.1 mg/kg/day to control the stiffness episodes. Developmental milestones were achieved as per age after two months of starting the therapy. The child is under regular follow-up.

## Conclusions

We reported a nine-month-old female patient with the potentially fatal but treatable neurological disease hyperekplexia, characterized by an exaggerated startle reflex and generalized stiffness since the neonatal period. Delay in the diagnosis of hyperekplexia, often due to incorrect diagnoses such as epilepsy or neuropsychiatric syndromes, as well as complex genetic neurodevelopmental disorders, exposes the child to unnecessary investigations, improper treatment, and financial burdens on the family. The assessment of the hyperekplexic startle response simply by nose tapping should be a routine part of examinations for all newborns. It highlights the critical need for immediate monitoring of at-risk infants, vigilant observation for signs of hyperekplexia, and ultimately, timely initiation of clonazepam in such patients.
